# Test your knowledge and understanding

**Published:** 2019-02-10

**This quiz is designed to help you test your own understanding of the concepts covered in this issue, and to reflect on what you have learnt.**

**Figure:**
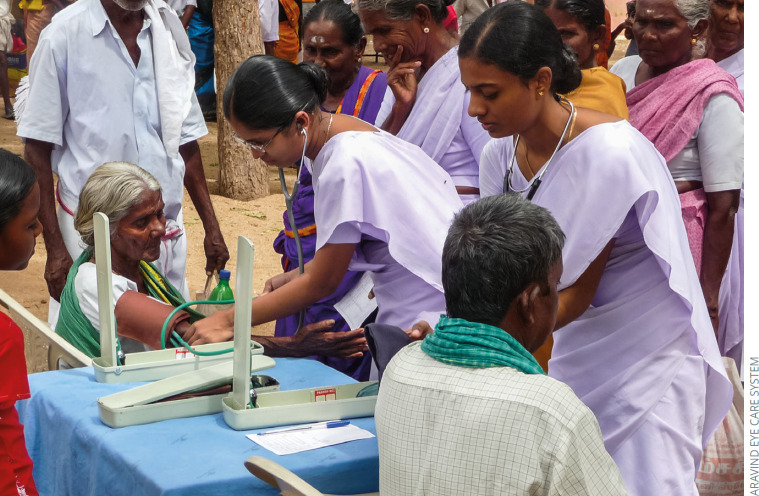
Measuring blood pressure in patients admitted for cataract surgery. **INDIA**

We hope that you will also discuss the questions with your colleagues and other members of the eye care team, perhaps in a journal club. To complete the activities online – and get instant feedback – please visit **www.cehjournal.org**

**Tick ALL that are TRUE**

**Table:** 

**Question 1** **When dealing with a patient with uveitis and cataract:**	
□	**a.** It does not matter if the uveitis is settled pre-operatively, so long as you give plenty of steroids postoperatively
□	**b.** Manipulating the iris to break synechiae or stretch a small pupil will increase the amount of postoperative inflammation
□	**c.** There is not much difference in the inflammatory response among the various types of IOL
□	**d.** It is a good idea to combine cataract surgery and glaucoma surgery if the patient has glaucoma
**Question 2** **In patients with diabetes who have cataract:**	
□	**a.** If the patient's sugar level is high on the day of surgery, it is better to delay the operation until their control is better
□	**b.** Postoperative capsular contraction can limit the view of the retina after surgery; therefore, a large capsulorhexis is advisable
□	**c.** Cataract starts younger in patients who have diabetes
□	**d.** Macular oedema is much more common after cataract surgery in patients who have diabetes
**Question 3** **If managing a patient with Fuch's endothelial corneal dystrophy:**	
□	**a.** It is quite easy to miss Fuch's endothelial corneal dystrophy in clinic when listing a patient for cataract surgery
□	**b.** Cohesive viscoelastics provide the best protection for the endothelium
□	**c.** If you don't have a specular microscope to count endothelial cells but can do pachymetry, you can use central corneal thickness as a marker of endothelial health
□	**d.** If you have access to phacoemulsification, then this is the best technique, even if you are not very experienced with it
**Question 4** **When operating on a patient with a small pupil:**	
□	**a.** Dilating drops should be put in 4–6 hours pre-operatively to give them maximum time to work
□	**b.** It is important to identify the cause of the small pupil pre-operatively
□	**c.** The options for managing small pupils are expensive, so additional charges will be required
□	**d.** If the pupil is dilated surgically, it may never return to its previous shape or size and the patient should be warned about this

## ANSWERS

1. a. False. It is essential to control the pre-operative inflammation before operating – preferably for 3 months or more. b. True. c. False. Different IOL materials promote varying levels of fibrotic response. d. False. In uveitis, combined surgery gives a very low success rate for the trabeculectomy.
2. a. False. Poor diabetic control has long term implications but will not affect the outcome of surgery. b. True. c. True. d. True.
3. a. True. b. False. Dispersive viscoelastics spread themselves onto the endothelium creating a protective coating. c. True. d. False. Effective surgery with the technique you are most familiar with is likely to minimise endothelial damage.
4. a. False. Dilation starts to wear off after a few hour. Starting dilating drops one prior to surgery is probably optimal. b. True. c. False. Bimanual pupil stretching should be adequate in most cases and involves no extra cost to the hospital other than slightly longer surgical time. d. True.

